# The Magnesium Membrane Shield Technique: A Structured and Simplified Approach for Severe Buccal Bone Deficiency in the Aesthetic Zones

**DOI:** 10.1002/ccr3.72443

**Published:** 2026-04-03

**Authors:** Giorgio Tabanella, Željka Perić Kačarević

**Affiliations:** ^1^ O.R.E.C. ‐ Oral Reconstruction and Education Center Rome Italy; ^2^ Department of Anatomy, Histology, Embriology, Pathology Anatomy and Pathology Histology, Faculty of Dental Medicine and Health Osijek J.J. Strossmayer University of Osijek Osijek Croatia

**Keywords:** aesthetic zone, dental implants, guided bone regeneration, magnesium membrane

## Abstract

Alveolar ridge preservation and regeneration remain critical challenges in implant dentistry and periodontics. This article presents a minimally invasive surgical approach for the management of complex buccal bone deficiencies using a novel biodegradable magnesium membrane. The Magnesium Membrane Shield Technique is designed to provide mechanical stability while gradually resorbing, thereby avoiding the need for secondary surgery and the additional morbidity associated with it. Its degradation involves the release of Mg^2+^ ions, which have been reported to modulate biological processes relevant to bone healing. The present study describes the biological rationale, clinical application, and radiographic observations associated with the treatment of a severe buccal plate loss case. Within the limitations of the presented case, this approach resulted in uneventful healing and satisfactory functional aesthetic outcomes, contributing to the existing clinical experience with magnesium‐based biomaterials in GBR compared with conventional resorbable and non‐resorbable approaches.

## Key Clinical Message

The Magnesium Membrane Shield Technique may offer a minimally invasive option for managing severe buccal bone defects following tooth extraction in the aesthetic area, while avoiding secondary surgery. Its resorbable yet structurally supportive properties, along with its potential biological effects, make it a promising alternative to conventional GBR approaches.

## 1 Introduction

Alveolar ridge preservation and regeneration remain critical challenges in implant dentistry and periodontics. After tooth extraction, alveolar bone resorption, especially on the buccal aspect, can compromise the volume and morphology of the ridge, leading to aesthetic and functional limitations. The buccal bone plate, typically thin and comprised mainly of bundle bone, is particularly vulnerable to rapid resorption following extraction, with reported reductions approaching 50% of the original thickness within the first six months [1]. This pronounced bone loss may lead to soft tissue collapse, impaired implant placement, and suboptimal aesthetic outcomes, especially in the anterior maxilla. Traditional methods for reconstructing alveolar defects include autogenous bone grafts, guided bone regeneration (GBR) with resorbable or non‐resorbable membranes, and socket preservation techniques aimed at mitigating ridge collapse. However, these approaches often involve multiple surgical steps, high morbidity, and limitations in achieving vertical bone regeneration [2, 3].

The emergence of bioresorbable magnesium membranes represents a significant advancement in regenerative dentistry. Unlike collagen or synthetic polymer membranes, magnesium‐based materials provide inherent mechanical strength and osteogenic potential. As magnesium degrades, it releases Mg^2+^ ions that stimulate osteoblast differentiation and enhance bone matrix formation [4, 5]. Additionally, the gradual resorption of the membrane eliminates the need for removal, reducing patient morbidity and simplifying treatment. The Magnesium Membrane Shield Technique, first described by Elad et al. [6], builds upon these advantages by combining the mechanical stability of magnesium with a minimally invasive clinical protocol for the single‐stage reconstruction of severe buccal plate deficiencies.

## 2 Clinical Case Presentation

**FIGURE 1:**
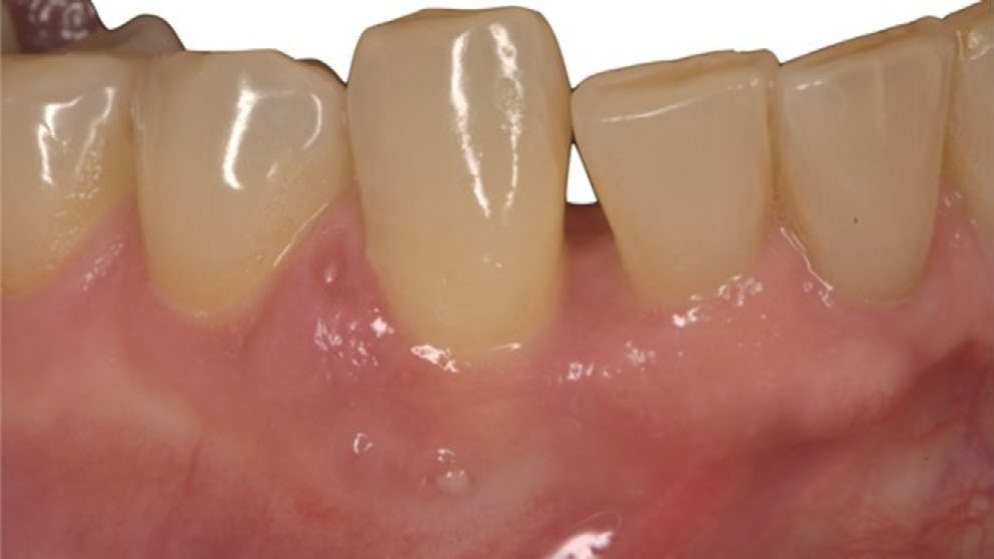
Clinical buccal view of the surgical site showing a mesial open contact and extrusion of the mandibular right canine. The gingival tissue presents a buccal fistula and signs of active abscess formation.

**FIGURE 2:**
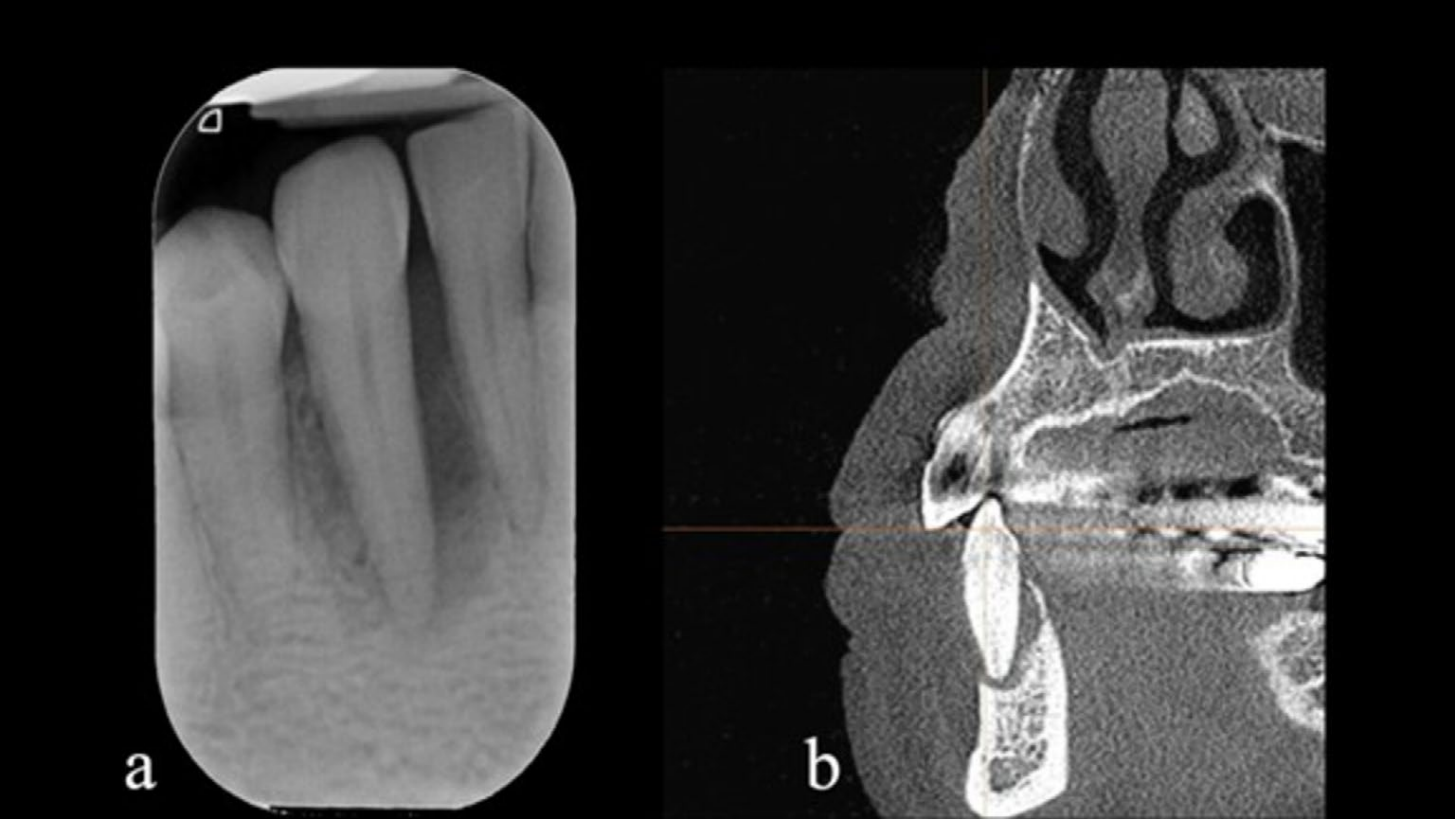
The periapical radiograph (a) and the corresponding sagittal CBCT section (b) display complete loss of the buccal bone plate and vertical bone loss exceeding beyond the root apex.

**FIGURE 3:**
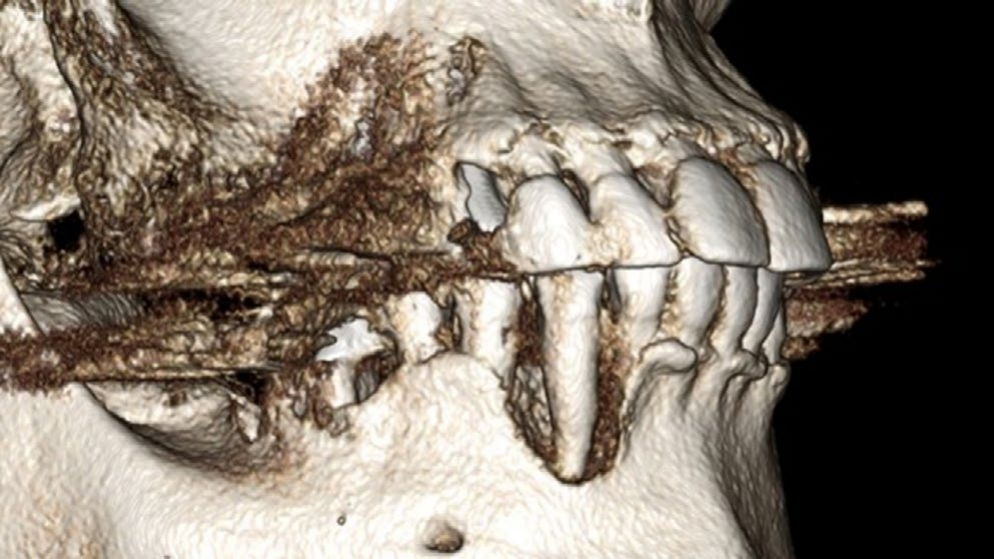
The 3D reconstruction shows a complex bony defect in an aesthetic area. Traditionally, such defects are managed by tooth extraction followed by a healing period of 3 months before performing guided bone regeneration (GBR), which is then followed by an additional healing phase of 8 months.

**FIGURE 4:**
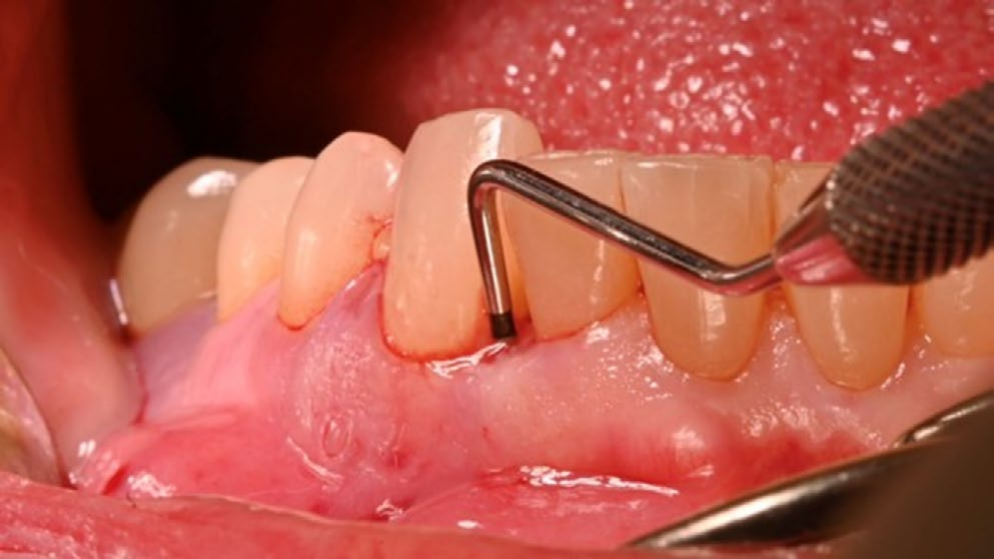
Periodontal probing depths exceeded 12 mm. Bleeding upon probing and suppuration are evident.

A 72‐year‐old female patient presented with advanced periodontal disease (Stage IV, Grade C) associated with suppuration and mobility class III of tooth #27 (Figures 1, 2, 3). No pathological probings were registered on the adjacent teeth which showed an intact periodontium as well as the absence of mucogingival deformities and mobility. Periodontal probing reported pocket depths ranging from 12 to 15 mm (Figure 4). The patient's medical history was non‐contributory except for smoking. A written informed consent was obtained previous surgical treatment.

## 3 Methods

### 3.1 Surgical Technique

**FIGURE 5:**
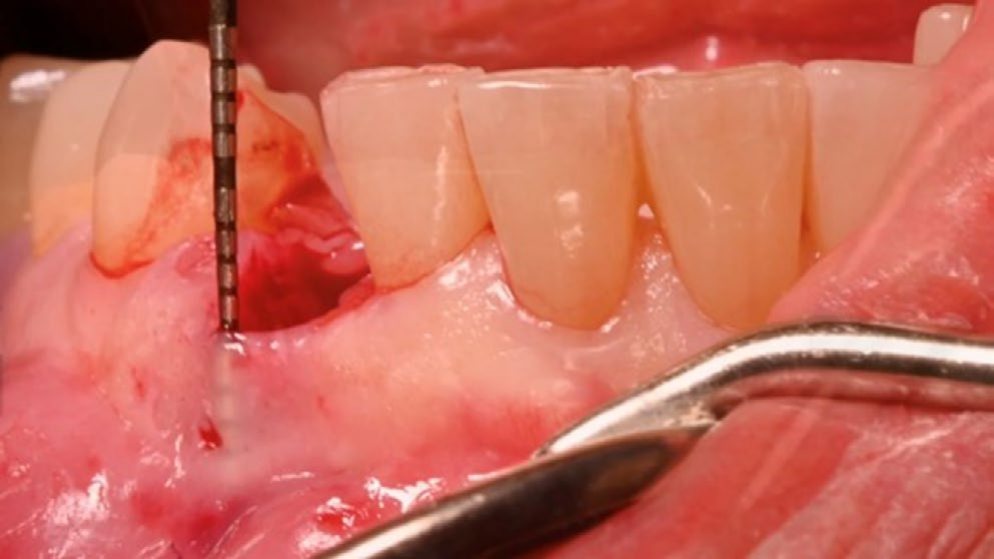
The buccal plate is completely resorbed, making the periodontal probe clearly visible through the soft tissue. The phenotype is thin.

The patient received premedication with 2 g of amoxicillin and clavulanic acid 1 h before surgery and continued with 1 g every 12 h for 5 days. After infiltration with local anesthesia (40‐mL solution of 4% articaine with adrenaline) an intrasulcular incision was performed around the mandibular right canine. The tooth was then gently extracted and removed (Figure 5). Careful degranulation of the inflamed tissue within the socket was carried out and an envelope full thickness flap was elevated without engaging the mesial and distal papilla of the neighboring healthy teeth.

### 3.2 Case Management

**FIGURE 6:**
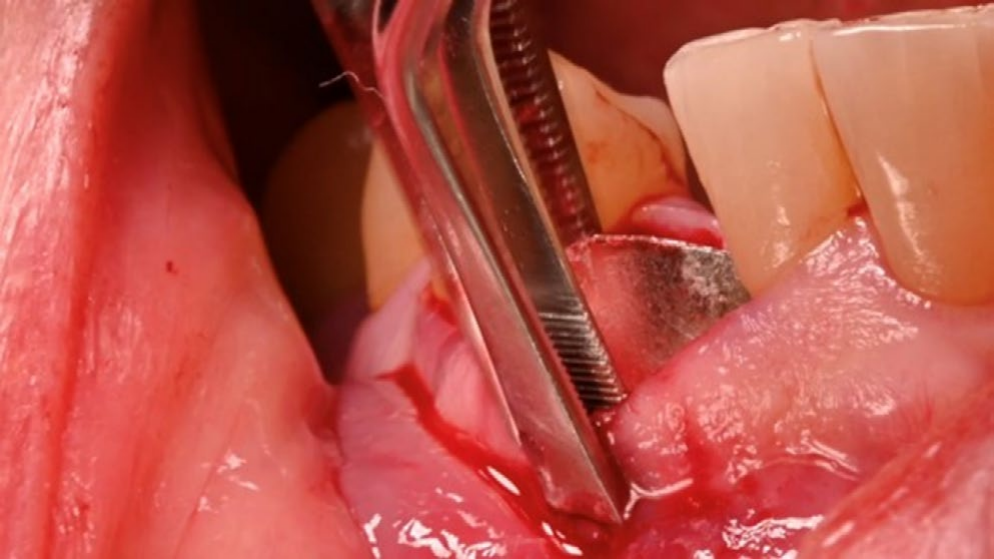
The Magnesium Membrane is trimmed and contoured to the defect morphology and inserted between the buccal flap and the residual vestibular bone.

**FIGURE 7:**
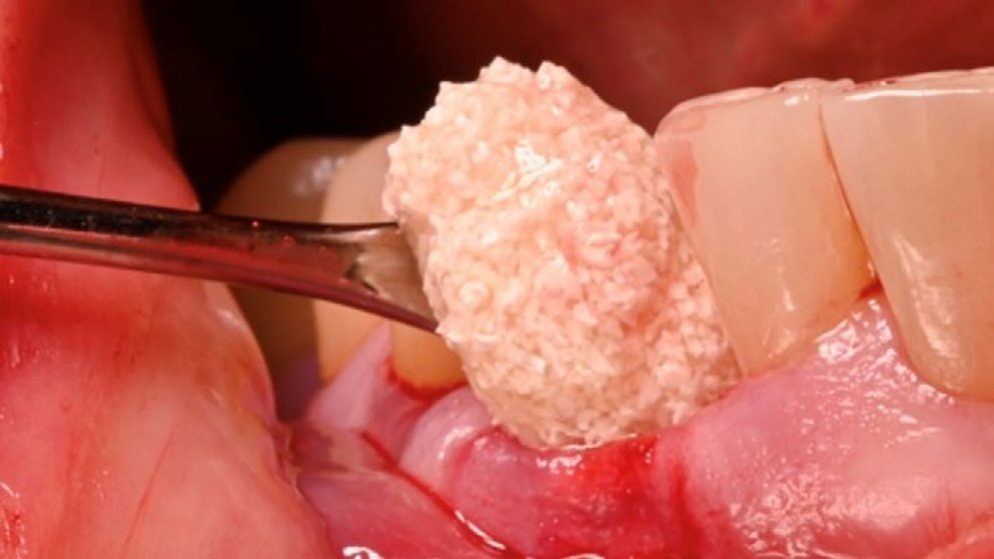
Demineralized bovine bone is condensed into the socket and compressed towards the membrane as well as mesially and distally. No additional fixation with pins or screws was used.

A trimmed magnesium membrane (NOVAMag membrane, Botiss biomaterials GmbH, Germany) was inserted between the elevated periosteum and the residual buccal plate (Figure 6). The membrane was positioned approximately 4 mm apical to the coronal portion of the vestibular bone and below the free mucosal margin to prevent exposure of the magnesium membrane to the oral cavity. Demineralized bovine bone (Cerabone plus Botiss biomaterials GmbH, Zossen, Germany) (Figure 7) was condensed into the socket and subsequently covered with spongious collagen (Collacone plus Botiss biomaterials GmbH, Zossen, Germany) to allow secondary intention healing. Crisscross sutures using asynthetic monofilament sutures were performed to provide pressure to the coronal portion of the socket.

## 4 Results

**FIGURE 8:**
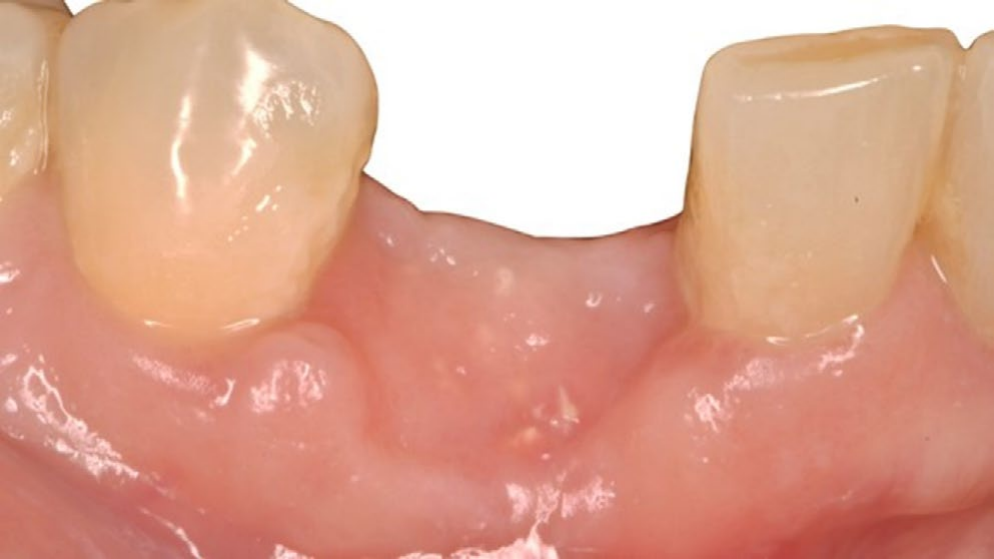
Clinical appearance 6 months postoperatively. The adjacent gingival margin remains stable without apical displacement.

**FIGURE 9:**
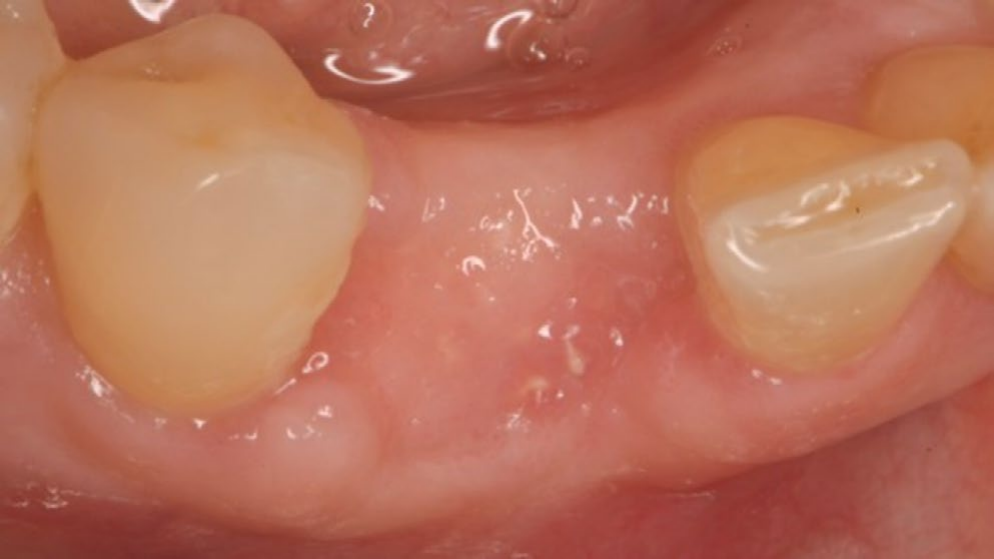
Secondary intention healing due to the open wound was associated with an adequate band of keratinized mucosa without additional soft tissue augmentation. The reconstructed buccal bone contour provides structural support to the overlying mucosal volume.

**FIGURE 10:**
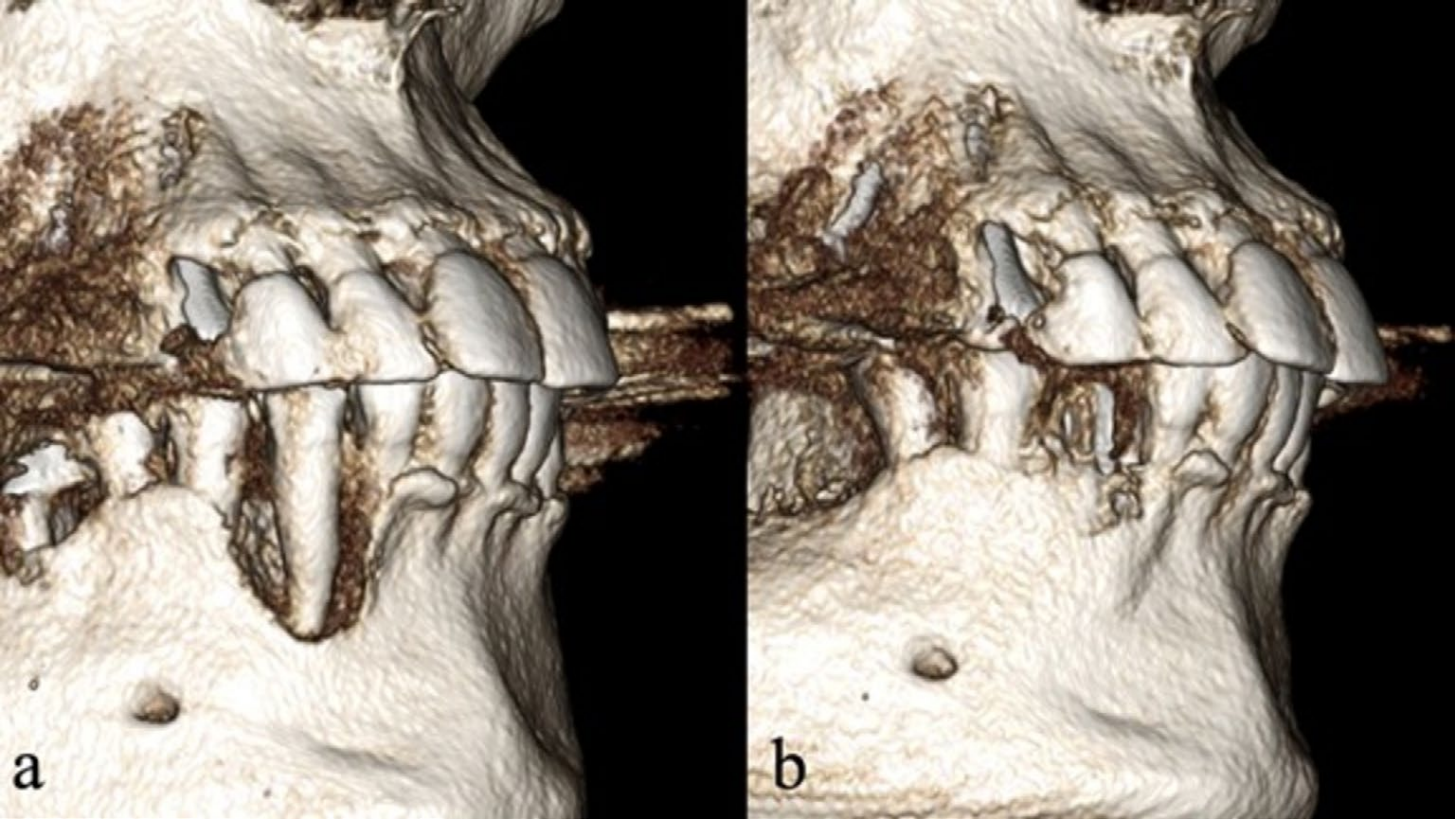
(a) Pre‐op 3D reconstruction demonstrating the buccal bone defect. (b) Six‐month postoperative 3D reconstruction following application of the Magnesium Shield Technique, showing reconstruction of the buccal contour.

**FIGURE 11:**
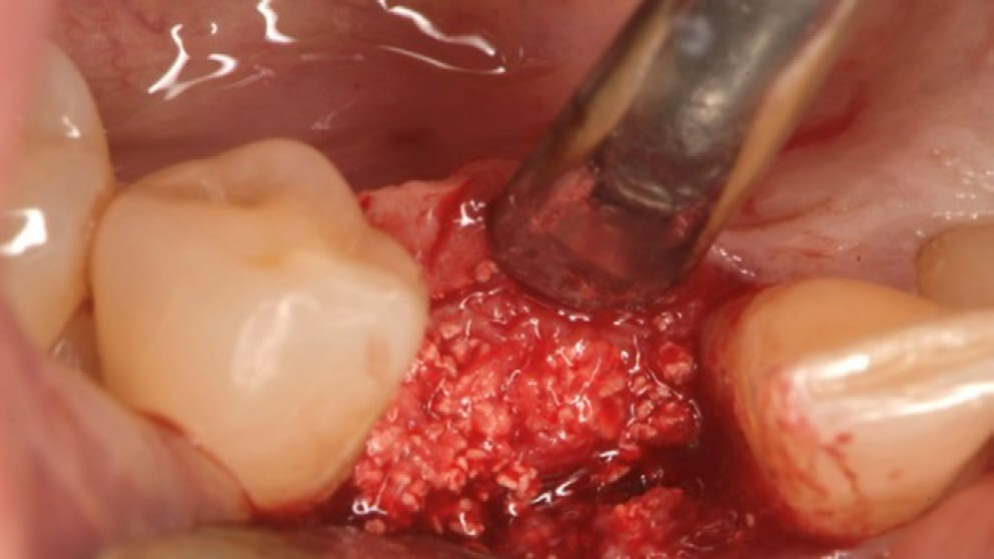
Regenerated bone volume after 6 months.

**FIGURE 12:**
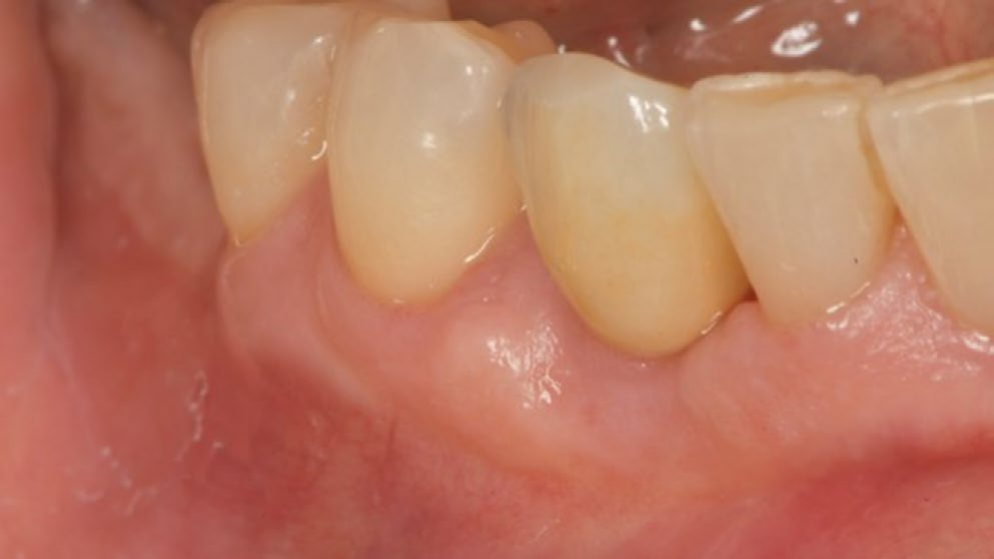
Follow‐up 1.5 years after delivery of the final restoration.

**FIGURE 13:**
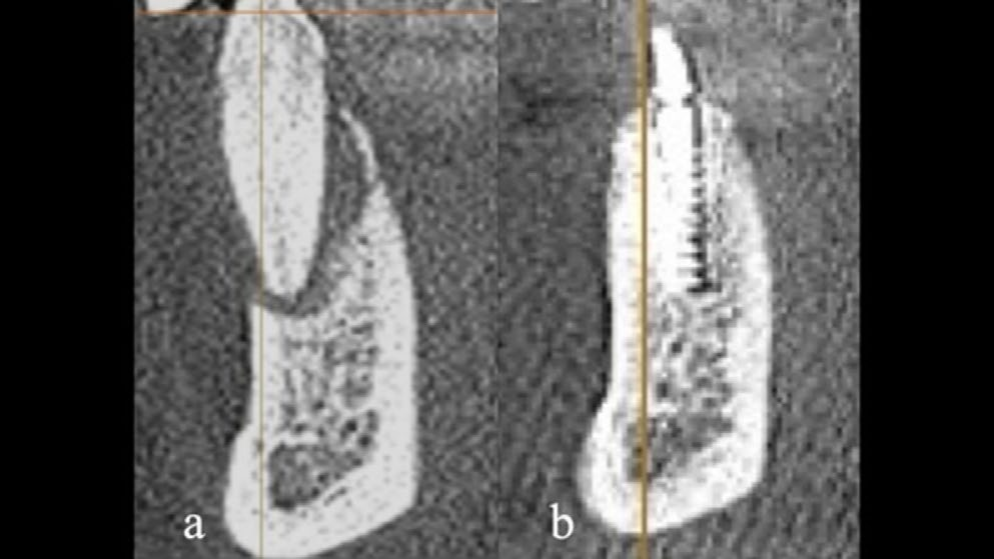
Sagittal sections: (a) Preoperative view before the application of the Shield Technique. (b) 1.5 years after delivery of the final restoration. The reconstructed buccal bone balcony appears maintained and stable.

After 6 months of uneventful healing (Figures 8 and 9), the 3D reconstruction clearly demonstrated vertical and horizontal augmentation (Figure 10), with a homogeneous radiographic appearance and increased bone density. A band of keratinized mucosa was visible following open wound healing (Figures 8 and 9). The adjacent dentition did not show any signs of attachment loss or gingival recession. After elevation of a full thickness flap (Figure 11) a dental implant was inserted achieving primary stability with an insertion torque exceeding 70Ncm. A healing period of two months was allowed for soft tissue maturation, after which a full ceramic CAD CAM screw retained crown was delivered following a provisional phase. At the 1.5‐year follow‐up after the delivery of the final restoration, a stable soft tissue collar and a favorable aesthetic outcome were observed (Figure 12). Radiographic evaluation demonstrated maintenance of the reconstructed buccal bone contour (Figure 13).

## 5 Discussion

The Magnesium Membrane Shield Technique represents an emerging approach building upon established principles of socket preservation and guided bone regeneration. Conventional non‐resorbable membranes such as expanded polytetrafluoroethylene (e‐PTFE) or titanium meshes, are known to provide reliable space maintenance; however, they typically require a second surgery for removal, which increases the risk of complications such as membrane exposure and infection [7, 8]. Resorbable collagen membranes eliminate the need for removal but lack rigidity, often collapsing under soft tissue pressure, particularly in vertical or combined defects [9]. Magnesium membranes, by contrast, are designed to maintain their structure during the critical healing phase while gradually resorbing in parallel with new bone formation. This dual behavior may provide stable space maintenance while potentially supporting bone regeneration.

During degradation, magnesium reacts with surrounding tissue fluids, releasing Mg^2+^ ions and forming a transient layer of magnesium hydroxide and hydrogen gas. The controlled release of Mg^2+^ ions has been shown to enhance osteogenic differentiation of mesenchymal stem cells by activating signaling pathways such as Wnt/β‐catenin and MAPK [10]. These ions also facilitate the deposition of calcium and phosphate, essential components of bone mineralization [11]. Simultaneously, the temporary formation of hydrogen gas bubbles has been described as potentially contributing to a mild tenting effect, maintaining the periosteal space and supporting the regenerative scaffold without compromising soft tissue healing [12].

In the presented case, the Magnesium Membrane Shield Technique was applied to a mandibular canine with advanced buccal and vertical bone loss of approximately 15 mm. After atraumatic extraction and debridement, the magnesium membrane was trimmed, contoured, and inserted on the buccal aspect as a substitute for the missing cortical plate. The defect was filled with demineralized bovine bone, and a collagen sponge was placed coronally to stabilize the clot and prevent particle migration. No flap elevation was performed, reducing surgical trauma and preserving blood supply. The magnesium membrane served as a self‐supporting barrier, maintaining the graft volume and guiding new bone formation. After 6 months, cone beam computed tomography (CBCT) revealed complete reconstruction of the buccal plate and sufficient bone volume for implant placement without the need for additional augmentation.

Histologic and radiographic findings from similar cases are consistent with the present observations. Studies by Elad et al. [6]. and Blasković et al. reported successful regeneration of cortical bone and favorable implant integration when magnesium membranes were used in GBR procedures [13]. In conjunction with these previously published reports, this case further documents the potential regenerative capacity associated with the Magnesium Membrane Shield Technique in the management of extensive vertical defects. Moreover, the controlled resorption profile of magnesium may provide sufficient barrier function during healing without prolonged presence that could interfere with remodeling.

A potential advantage of magnesium membranes compared with conventional barrier membranes relates to their degradation behavior and associated bioactive properties. While collagen membranes primarily function as passive barrier materials, magnesium‐based membranes release Mg^2+^ ions during degradation, which have been associated with modulation of osteogenic pathways [14]. Experimental studies demonstrate that Mg^2+^ concentrations in the physiological range upregulate alkaline phosphatase activity, osteocalcin expression, and collagen synthesis in osteoblasts [14, 15]. This biological stimulation not only accelerates new bone formation but also enhances the quality of regenerated bone, leading to a denser and more stable cortical architecture. In this context, a review comparing vertical augmentation strategies in large ridge defects reported that less invasive approaches such as computer‐aided design/computer‐aided (CAD/CAM)‐fabricated titanium meshes, the Magnesium Shield Technique, and the Shell Technique using allogenic plates were associated with shorter surgical times, lower complication rates, and lower biological costs compared with iliac crest grafting, which has traditionally been considered an established reference approach for the reconstruction of severe vertical ridge defects, while still achieving substantial vertical bone gains exceeding 3 mm [16].

Despite these advantages, the clinical use of magnesium membranes requires attention to specific considerations. Rapid degradation or hydrogen gas accumulation may occur if the membrane is exposed to saliva or inflammatory fluids [17]. Therefore, primary wound closure and proper flap management are considered essential. Early studies reported mild transient inflammation around degrading magnesium implants; however, these reactions were self‐limiting and did not compromise healing [18]. Clinical trials have confirmed that magnesium membranes exhibit comparable safety to collagen membranes and show high implant survival rates after GBR procedures [19]. Nevertheless, as the present report describes a single clinical case, these findings should be interpreted with caution.

Taken together, The Magnesium Membrane Shield Technique offers a balanced combination between mechanical and biological performance. It enables single‐stage reconstruction of buccal bone deficiencies, potentially reducing the need for block grafts or titanium meshes. Compared with autogenous block grafting—which requires a donor site and carries risks of resorption and morbidity [20]—the magnesium membrane offers an off‐the‐shelf alternative with clinically favorable outcomes. Furthermore, the combined effects of space maintenance and potential osteogenic signaling may support the regenerative process while potentially contributing to a more streamlined treatment timeline.

Future research should focus on optimizing magnesium membrane design through alloy composition and surface modification. Adjusting degradation kinetics and incorporating bioactive coatings or antimicrobial agents may further enhance the membrane performance. Recent preclinical studies indicate that magnesium‐based scaffolds can be functionalized with growth factors such as BMP‐2 or VEGF to stimulate angiogenesis and bone maturation [21]. Such developments may expand the clinical applications of magnesium‐based GBR beyond socket preservation to include ridge augmentation, sinus floor elevation, and peri‐implant defect management.

## 6 Conclusion

Within the limitations of the presented case, The Magnesium Membrane Shield Technique was associated with stable hard‐ and soft‐tissue healing in the reconstruction of a severe buccal plate deficiency in an aesthetic area. The membrane's mechanical stability, biodegradability, and potential biological activity may have contributed to an ideal environment for bone regeneration while avoiding some limitations of conventional GBR materials. When considering alongside existing clinical data, these findings support the membrane's ability to achieve favorable functional and aesthetic outcomes within a single surgical stage, reducing patient morbidity and overall treatment time. However, further investigation into magnesium‐based biomaterials is required to better understand their role as a transformative solution in guided bone regeneration.

## Author Contributions

**Giorgio Tabanella:** conceptualization, investigation, methodology, writing – original draft. **Željka Perić Kačarević:** supervision, writing – review and editing.

## Funding

This study was supported by this case report received no external funding.

## Ethics Statement

According to institutional and national guidelines, ethics approval was not required for this case report.

## Consent

Written informed consent was obtained from the patient for publication of this case report and images.

## Conflicts of Interest

The authors declare no conflicts of interest.

## Data Availability

All data generated or analyzed during this study are included in this published article. No addi‐tional datasets were created or analyzed.
