# Correction to “The Magnesium Membrane Shield Technique: A Structured and Simplified Approach for Severe Buccal Bone Deficiency in the Aesthetic Zones”

**DOI:** 10.1002/ccr3.73110

**Published:** 2026-07-13

G. Tabanella and Ž. P. Kačarević , “The Magnesium Membrane Shield Technique: A Structured and Simplified Approach for Severe Buccal Bone Deficiency in the Aesthetic Zones,” Clinical Case Reports 14, no. 4 (2026): e72443, 10.1002/ccr3.72443.41948754 PMC13052211

In Reference 3, the bibliographic information was incorrect. The corrected reference is provided below:

Soldatos NK, Stylianou P, Koidou VP, Angelov N, Yukna R, Romanos GE. Limitations and options using resorbable versus nonresorbable membranes for successful guided bone regeneration. *Quintessence International*. 2017;48(2):131–147. https://doi.org/10.3290/j.qi.a37133.

We apologize for this error.

